# Hyperdense Basal Ganglia on Brain CT in Non-Ketotic Hyperglycemia Associated with Hemichorea

**DOI:** 10.5334/jbsr.2706

**Published:** 2022-04-29

**Authors:** Bjorn Valgaeren, Elyn Van Snick, Jan Hendrickx

**Affiliations:** 1 General Hospital Damiaan Ostend, BE

**Keywords:** hemichorea, non-ketotic hyperglycemia, brain CT, diabetes mellitus, lentiform nucleus, caudate nucleus

## Abstract

**Teaching Point:** Unilateral choreiform movements combined with contralateral hyperdense lentiform and/or caudate nucleus on computed tomography is suggestive for non-ketotic hyperglycemia, warranting further metabolic workup.

## Case History

**Figure 1:**
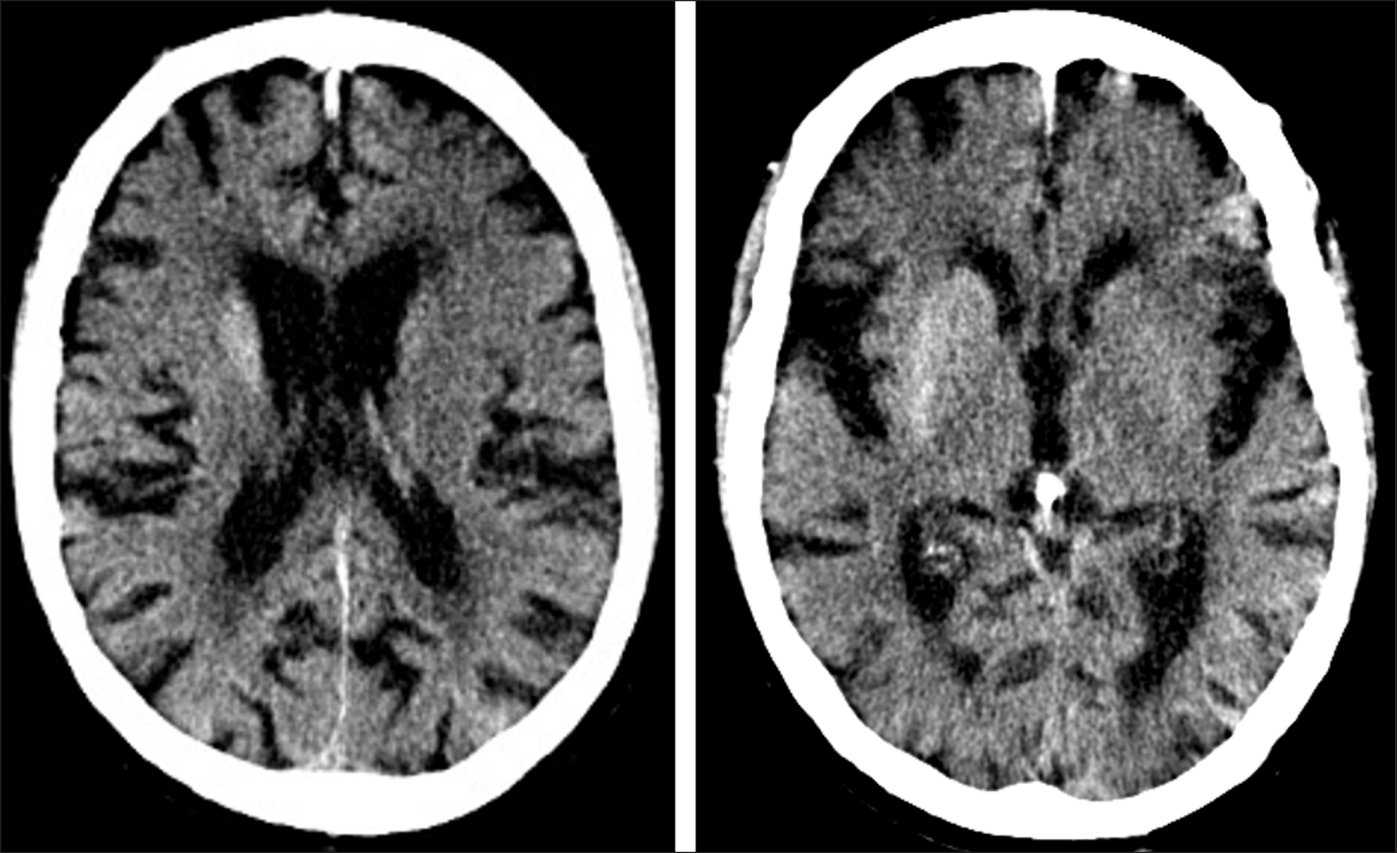


An 88-year-old male with dementia was sent to the emergency department for bilateral choreiform movements and worsening confusion. Brain computed tomography (CT) was performed, showing a hyperdense lentiform nucleus, more prominent on the right side than on the left side. A hyperdense right-sided caudate nucleus with 52 Hounsfield units (HU) versus 42 HU (***Figure 1***). Noticeable cerebral atrophy, old lacunar supratentorial infarctions, and periventricular leukoencephalopathy hadn’t changed significantly compared to a prior examination one year earlier.

Hemoglobin A1c (HbA1c) and blood glucose level were measured. The glycemia was normal, but high HbA1c of 13.8% or 127 mmol/mol (normal 4–6% or 20–42mmol/mol) confirmed the diagnosis of new onset diabetes mellitus. During the work-up for the diabetes, abdominal CT demonstrated a mass in the body of the pancreas, believed to be the cause of the diabetes mellitus. Considering the patient’s mental state, no further investigation was performed.

## Comment

Hemichorea consists of unilateral, continuous, nonrhythmic, rapid, involuntary dance-like movements [1]. When related to non-ketotic hyperglycemia,

CT findings are usually unilateral hyperdense striatum on the contralateral side of the choreiform movements, but can be bilateral [1].

The most important magnetic resonance imaging (MRI) findings are unilateral high signal in the affected caudate and/or lentiform nucleus on T1-weighted images, increased paramagnetic susceptibility and mild to moderate diffusion restriction [1]. Although the exact pathophysiology is unknown, it is believed that in non-ketotic hyperglycemia, the striatum suffers acute ischemic injury which leads to reactive swelling and recruitment of astrocytes, known as gemistocytes. These damaged cells contain high intracellular water content responsible for restricted diffusion. The gemistocytes also contain a high concentration of zinc-friendly metalloproteins, iron and copper, increasing paramagnetic susceptibility and CT density. The characteristic prominent protein hydration layer of these cells, and to a lesser extent the metal ions, are responsible for the shortened T1 relaxation time [1].

If found incidentally, these findings warrant further investigation of blood glucose levels, serum osmolality and urinary ketone levels. High blood glucose levels and elevated serum osmolality are usually found, whereas urinary ketone levels are normal [1]. Treatment involves checking and normalizing blood glucose levels with intravenous insulin therapy. Additional symptom management can be achieved by administering dopamine receptor antagonists, for example, haloperidol. Generally, patients have a good prognosis after achieving acceptable blood glucose levels and imaging findings will slowly resolve back to normal [1].
